# Supplementary material for: Bayesian refinement of protein structures and ensembles against SAXS data using molecular dynamics
Source: PLoS Comput Biol. 2017 Oct 18;13(10):e1005800. doi: 10.1371/journal.pcbi.1005800 (PMC5662244; doi:10.1371/journal.pcbi.1005800)
Supplement: S1 Archive — (BZ2) [file pcbi.1005800.s011.tar.bz2 › gromacs_4.62_bayesian_saxs_refi/share/html/online/g_hydorder.html]

g\_hydorder


| g\_hydorder Main Table of Contents |

**VERSION 4.6  
Sat 19 Jan 2013**

---

### Description

g\_hydorder computes the tetrahedrality order parameters around a
given atom. Both angle an distance order parameters are calculated. See
P.-L. Chau and A.J. Hardwick, Mol. Phys., 93, (1998), 511-518.
for more details.  
This application calculates the orderparameter in a 3d-mesh in the box, and
with 2 phases in the box gives the user the option to define a 2D interface in time
separating the faces by specifying parameters -sgang1 and -sgang2 (It is important
to select these judiciously)

### Files

| option | filename | type | description |
| --- | --- | --- | --- |
| **-f** | traj.xtc | Input | Trajectory: xtc trr trj gro g96 pdb cpt |
| **-n** | index.ndx | Input | Index file |
| **-s** | topol.tpr | Input | Run input file: tpr tpb tpa |
| **-o** | intf.xpm | Output, Mult. | X PixMap compatible matrix file |
| **-or** | raw.out | Output, Opt., Mult. | Generic output file |
| **-Spect** | intfspect.out | Output, Opt., Mult. | Generic output file |

### Other options

| option | type | default | description |
| --- | --- | --- | --- |
| **-[no]h** | bool | no | Print help info and quit || **-[no]version** | bool | no | Print version info and quit || **-nice** | int | 19 | Set the nicelevel || **-b** | time | 0 | First frame (ps) to read from trajectory || **-e** | time | 0 | Last frame (ps) to read from trajectory || **-dt** | time | 0 | Only use frame when t MOD dt = first time (ps) || **-[no]w** | bool | no | View output .xvg, .xpm, .eps and .pdb files || **-d** | enum | z | Direction of the normal on the membrane: z, x or y || **-bw** | real | 1 | Binwidth of box mesh || **-sgang1** | real | 1 | tetrahedral angle parameter in Phase 1 (bulk) || **-sgang2** | real | 1 | tetrahedral angle parameter in Phase 2 (bulk) || **-tblock** | int | 1 | Number of frames in one time-block average || **-nlevel** | int | 100 | Number of Height levels in 2D - XPixMaps |

---

http://www.gromacs.org  
gromacs@gromacs.org
